# Superadiabatic Forces via the Acceleration Gradient in Quantum Many-Body Dynamics

**DOI:** 10.3390/molecules24203660

**Published:** 2019-10-11

**Authors:** Moritz Brütting, Thomas Trepl, Daniel de las Heras, Matthias Schmidt

**Affiliations:** Theoretische Physik II, Physikalisches Institut, Universitat Bayreuth, D-95440 Bayreuth, Germany

**Keywords:** superadiabatic effects, density functional theory, time-dependent density functional theory, Hooke’s atom, power functional theory

## Abstract

We apply the formally exact quantum power functional framework (*J. Chem. Phys.* **2015**, *143*, 174108) to a one-dimensional Hooke’s helium model atom. The physical dynamics are described on the one-body level beyond the density-based adiabatic approximation. We show that gradients of both the microscopic velocity and acceleration field are required to correctly describe the effects due to interparticle interactions. We validate the proposed analytical forms of the superadiabatic force and transport contributions by comparison to one-body data from exact numerical solution of the Schrödinger equation. Superadiabatic contributions beyond the adiabatic approximation are important in the dynamics and they include effective dissipation.

## 1. Introduction

While the dynamical behaviour of a non-relativistic quantum many-particle system is given by the Schrödinger equation, obtaining in practice the time-dependent wave function by direct solution often comes at prohibitive computational cost. A reformulation on the one-body level helps to systematically approximate the physical behaviour and to gain deeper insights into the fundamental physical processes at play. For groundstate properties, density functional theory (DFT) [1] provides such formally exact reformulation, see reference [2] for a recent account of DFT. In DFT the external potential is established as a unique functional of the one-body density. The DFT framework has been extended to include both the current density and the spin density in order to describe groundstate properties of inhomogeneous electronic systems in magnetic fields [3,4].

The Runge-Gross theorem [5] forms the basis of *time-dependent* DFT; see, e.g., references [6,7,8] for recent accounts. In subsequent fundamental work Vignale and Kohn [9] introduced the current density as the basic variable in a generalized time-dependent Kohn-Sham scheme. Vignale, Ullrich and Conti [10] went beyond the adiabatic local density approximation. In their formulation, dynamical exchange and correlation effects appear as viscoelastic stresses in the electron fluid. Ghosh and Dhara [11] had originally developed a time-dependent DFT scheme for arbitrary time dependence. Vignale [12] went beyond their work in formulating a mapping from current densities to vector potentials in time-dependent current DFT. In his general case, the functional map is onto a system with a different two-particle interaction potential. The Runge-Gross theorem is then obtained as a mere special case, see reference [12]. In typical applications one is often limited to the adiabatic approximation, i.e., using an instantaneous corresponding groundstate as a means to investigate non-equilibrium phenomena [6,7,13,14,15]. While much research effort has been directed to overcome this limitation [16,17,18,19,20,21,22,23], there is no clear consensus on how to go beyond the adiabatic approximation. Reference [24] presents an in-depth comparison of two primary approaches, i.e., time-dependent current DFT [9,10] and Tokatly’s Lagrangian quantum fluid mechanics approach [16,17,18]. In reference [22] Ullrich applies and scrutinizes the former framework for charge oscillations in quantum strip with two electrons.

The situation in the classical realm is similar: Classical density functional theory [25] provides a formally exact framework for the finite-temperature statistical physics of (classical) liquids and solids. The dynamical extension to overdamped Brownian dynamics [25,26,27] is an adiabatic approximation restricted to forces that can be derived from the equilibrium free energy functional. However, recent work has established power functional theory as a formally exact variational framework for capturing the genuine non-equilibrium (“superadiabatic”) effects [28] in Brownian dynamics. Here both the density and the current profiles act as the central variables. The one-body internal force field is systematically split into an adiabatic and a superadiabatic contribution [28,29,30]. Based on the gradient of the velocity field, approximations of the superadiabatic forces were systematically constructed, and shown to describe both viscous [31] and structural effects [32].

For overdamped Brownian dynamics, the (power) functional [28] that generates the exact equation of motion is technically a current-density functional, similar in mathematical structure to what is used in the description of quantum systems based on current-density-functional theory [3,4,9,10,12]. However, the role of the one-body current in overdamped (classical) motion is very different from that in the inertial (quantum) case. For Hamiltonian dynamics, the exact power functional minimization is rather with respect to the time derivative of the current, as has been shown both for classical Newtonian many-body dynamics [33] and for (time-dependent) quantum systems [34].

Investigating model systems with two electrons is a common means for addressing fundamental questions in time-dependent DFT, such as, e.g., the occurrence of dynamical step structures in the time-dependent exchange–correlation potential [35], initial-state dependence [36], the sign of the time-dependent correlation energy [23], the exact exchange potential [37] (as obtained via the time-dependent optimized effective potential method [38,39,40]), an exact condition for time-resolved spectroscopy [41], the kinetic and interaction components of the exact time-dependent correlation potential [42], the dynamics of charge-transfer processes [43], field-induced tunneling [44] and Rabi oscillations [45].

In this paper we demonstrate the validity of the quantum power functional theory (PFT) [34] using a simple one-dimensional Hooke’s atom (see e.g., [23]) consisting of two electrons interacting via a repulsive Gaussian pair potential. We demonstrate that in a time-dependent situation (switching off the confining “nuclear” harmonic potential) three types of superadiabatic contributions occur: (i) A transport term due to an excess kinetic stress tensor contribution, (ii) a volume viscous force and (iii) a genuine inertial force contribution. We demonstrate that all three one-body force contributions can be represented as functionals of the velocity gradient and of the acceleration gradient.

## 2. Theoretical Background

Quantum PFT is formulated for Hamiltonians of the form

(1) $$\hat{H}=\sum_{i}\frac{{\hat{p}}_{i}^{2}}{2m}+u\left(r^{N}\right)+\sum_{i}V_{\mathrm{ext}}\left(r_{i},t\right),$$

where ${\hat{p}}_{i}$ is the kinematic momentum operator of particle *i* with mass *m*, $u(r^{N})$ is the many-body interparticle interaction, which depends in general on the set of all *N* particle positions $r^{N}\equiv \left\{r_{1}\cdots r_{N}\right\}$, and $V_{\mathrm{ext}}\left(r_{i},t\right)$ is the external potential acting on particle *i* at time *t*. Quantum PFT [34] is based on the current J and its time derivative $\dot{J}$ as further central variables besides the density *n*. For the physical dynamics the one-body distributions are given by $n=\langle \sum_{i}\delta_{i}\rangle$, $J=\langle \sum_{i}\left(\delta_{i}{\hat{p}}_{i}+{\hat{p}}_{i}\delta_{i}\right)/2m\rangle$, and $\dot{J}=dJ/dt$, where $\delta_{i}\equiv \delta \left(r-r_{i}\right)$ is the Dirac distribution, r indicates position and 〈·〉 denotes the quantum mechanical average. The density profile and the current distribution are related by the continuity equation

(2) $$\dot{n}=-\nabla \cdot J,$$

where ∇ indicates the derivative with respect to r. Hence it takes two time integrals to obtain *n* once $\dot{J}$ is known; we suppress the dependence on r,t in the notation.

The central object of quantum PFT is the power rate functional $G_{t}\left[n,J,\dot{J}\right]$ which is minimized by that specific function $\dot{J}$ at time *t* that corresponds to the real physical dynamics. The exact one-body equation of motion connects $\dot{J}$ to the total force density [46,47] (As shown in [34], Equation (3) can also be derived on the level of the corresponding single particle operators from the Heisenberg equation of motion, i.e., from the generic Hamiltonian Equation (1). Thereby, also magnetic fields can be captured, though excluded here for simplicity).

(3) $$m\dot{J}=\nabla \cdot \tau +\frac{\hbar^{2}}{4m}\Delta \nabla n-n\nabla V_{\mathrm{ext}}+F_{\mathrm{int}}.$$

Here $\Delta \equiv \nabla^{2}$ is the Laplace operator, and *ℏ* indicates the reduced Planck constant. The first and second terms on the right hand side of Equation (3) describe transport contributions which arise from the one-body description; τ is the local kinetic stress tensor (or one-body momentum current) given by

(4) $$\tau \left(r,t\right)\equiv -\langle \sum_{i}\frac{{\hat{p}}_{i}\delta_{i}{\hat{p}}_{i}+{\left({\hat{p}}_{i}\delta_{i}{\hat{p}}_{i}\right)}^{T}}{2m}\rangle ,$$

where the superscript T indicates matrix transposition.

The third term in Equation (3) represents the external force density generated by the external potential $V_{\mathrm{ext}}\left(r,t\right)$; the fourth term is the internal force density $F_{\mathrm{int}}\left(r,t\right)\equiv -\langle \sum_{i}\left(\nabla_{i}u\right)\delta_{i}\rangle$ due to the interparticle interaction potential $u(r^{N})$. In the PFT framework, Equation (3) is obtained from minimization of the power rate functional $G_{t}\left[n,J,\dot{J}\right]$ with respect to the time derivative of the current, $\dot{J}\left(r,t\right)$. The minimization is performed in a “time slice” at time *t*.

Equations (2) and (3) form a closed and formally exact set of equations which governs the dynamics of *n* and J, as quantum PFT guarantees that τ and $F_{\mathrm{int}}$ can be expressed as functionals of *n*, J and $\dot{J}$. Hence as soon as $F_{\mathrm{int}}$ and τ are given as, say, approximate functionals of n,J and $\dot{J}$, then one can integrate in time Equations (2) and (3), together expressing J as the time integral of $\dot{J}$. If the spatial part of the wave function is symmetric under particle exchange, and the interparticle interaction potential vanishes, then the kinetic stress tensor reduces to its ideal form [34]

(5) $$\tau_{\mathrm{id}}=-m\frac{JJ}{n}-\frac{\hbar^{2}}{4m}\frac{\left(\nabla n)(\nabla n\right)}{n}.$$

In general, additional contributions arise from (i) the interparticle interaction and (ii) for fermionic systems also from the exchange interaction due to the antisymmetry of the wave function. These effects are encapsulated in the excess stress contribution

(6) $$\tau_{\mathrm{exc}}\equiv \tau -\tau_{\mathrm{id}}.$$

The internal force density splits into adiabatic and superadiabatic contributions:

(7) $$F_{\mathrm{int}}=F_{\mathrm{ad}}+F_{\sup},$$

where the adiabatic force density $F_{\mathrm{ad}}$ at time *t* is defined as the internal force density of a hypothetical (“adiabatic”) ground state system, which has the same one-body density as the real system at time *t* [14]. Within the adiabatic approximation $F_{\sup}$ is neglected. Here we show how to overcome this restriction.

## 3. Hooke’s Atom

We study a Hooke’s model atom [23] with N=2 electrons in one spatial dimension, which reduces the numerical effort of solving the Schrödinger equation. The interparticle interaction is modelled by a repulsive soft-core potential $\phi \left(x_{1}-x_{2}\right)=\epsilon \exp\left(-{\left(x_{1}-x_{2}\right)}^{2}/2\alpha^{2}\right)$, where $x_{1}-x_{2}$ is the interparticle distance, ϵ≥0 the strength and α the length-scale of the repulsion. The initial state (t=0) is chosen as the ground state in the external (“nuclear”) potential $V_{\mathrm{ext}}\left(x\right)=m\omega^{2}x^{2}/2$, i.e., a harmonic oscillator with frequency ω; here *x* indicates position. We consider the two electrons to be of opposite spin. The spin part of the wave function is antisymmetric under particle exchange, and hence the spatial part is symmetric. At time t=0 we switch off $V_{\mathrm{ext}}$ and monitor the resulting time evolution.

We determine the initial wave function by minimizing the energy expectation value $\langle \hat{H}\rangle$. Next, we solve the time-dependent Schrödinger equation by an explicit integration scheme [48,49] based on discretizing the time evolution operator. The adiabatic system can be constructed explicitly [14] by numerically finding the suitable external potential that produces the desired one body density. Such potential is unique according to Hohenberg and Kohn [1]. We find the adiabatic potential using the method of references [29,30], originally developed for Brownian dynamics (see reference [50] for an alternative method). The potential that generates a given target density profile is found by iteratively improving an initial guess. At each iteration the density profile follows from solving the time-independent Schrödinger equation. The method converges towards the target profile.

**Figure 1:**
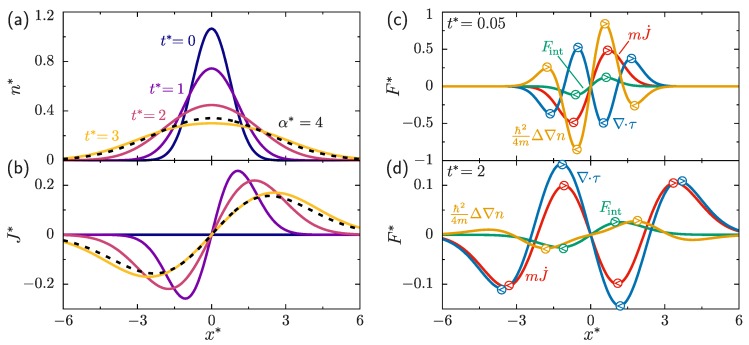
Time evolution of (**a**) the reduced density $n^{*}=n\sqrt{\hbar /m\omega }$ and (**b**) the reduced current density $J^{*}=J/\omega$ as a function of the reduced box coordinate $x^{*}=x\sqrt{m\omega /\hbar }$ for ϵ/ℏω=0.5 and $\alpha^{*}=1$. For comparison, at reduced time $t^{*}\equiv t\omega =3$ the curves for $\alpha^{*}=4$ are also shown (dashed line). Illustration of the total force density and the different contributions according to Equation (3) for $t^{*}=0.05$ (**c**) and $t^{*}=2$ (**d**) given in reduced units as $F^{*}=F/m\omega^{2}$. The encircled arrows indicate the force direction at selected space points. The total size of the box is $30\sqrt{\hbar /m\omega }$.

An overview of the dynamics is given in Figure 1. The one-body density, Figure 1a, possesses initially a sharp peak which broadens after switching off $V_{\mathrm{ext}}$. The current, Figure 1b, points away from the center of the density peak. For $t^{*}\equiv t\omega =3$ we show *n* and J for two different interaction lengths, $\alpha^{*}=\alpha \sqrt{m\omega /\hbar }=1$ and 4. The differences between both cases are relatively small. The dynamics are driven by the different terms on the right hand side of Equation (3); corresponding results for each of these terms are shown at times $t^{*}=0.05$ and $t^{*}=2$ in Figure 1c,d, respectively. At early times the dynamics are dominated by contributions proportional to $\hbar^{2}$ which arise from spatial variations from the density and are also referred to as the non-interacting quantum stress tensor [15]. For later times the contribution of $\nabla \cdot \tau_{\mathrm{id}}$ to the current is the most prominent one. The internal force density is relevant at both early and late times.

We next analyse the stress tensor and the internal force density. We perform the splittings in Equations (6) and (7) and use the data as a guide to formulate approximate forms for the functionals $\tau_{\mathrm{exc}}$ and $F_{\sup}$. The mathematical structure of the proposed functional forms [as given below in Equations (8), (10) and (11)] is inspired by studies of classical systems [31,32], where superadiabatic forces in overdamped Brownian dynamics are described by an expansion in terms of the velocity gradient. Within the framework of quantum PFT also the acceleration field is relevant. In one dimension, the velocity and the acceleration fields are defined, respectively, as v(x,t)=J/n and $a\left(x,t\right)=\dot{J}/n$. Hence, our goal is to express $\tau_{\mathrm{exc}}$ and $F_{\sup}$ as functionals of n,v and *a*.

**Figure 2:**
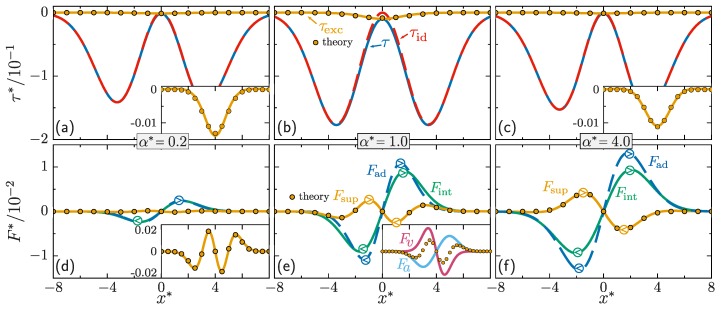
Reduced kinetic stress tensor $\tau^{*}=\tau /\sqrt{m\omega^{3}\hbar }$ (blue solid line) and its splitting into ideal (red dashed line) and excess (solid yellow line) parts, Equation (6), and the theoretical prediction (yellow circles), Equation (8), for interaction lengths $\alpha^{*}=0.2$ (**a**), $\alpha^{*}=1$ (**b**) and $\alpha^{*}=4$ (**c**). Internal (green solid line), adiabatic (blue dashed line) and superadiabatic (yellow solid line) force densities, cf. Equation (7), and theoretical prediction (yellow circles), Equation (9), in reduced units, i.e., $F^{*}=F/m\omega^{2}$ for interaction lengths $\alpha^{*}=0.2$ (**d**), $\alpha^{*}=1$ (**e**) and $\alpha^{*}=4$ (**f**). Data taken at $t^{*}=3$. The model parameters and reduced units are identical to those in Figure 1. The insets in panels (**a**,**c**,**d**) are enlarged views of the superadiabatic terms. The inset in panel (**e**) shows the splitting of the superadiabatic force density (circles) into $F_{v}$ (pink) and $F_{a}$ (blue).

**Figure 3:**
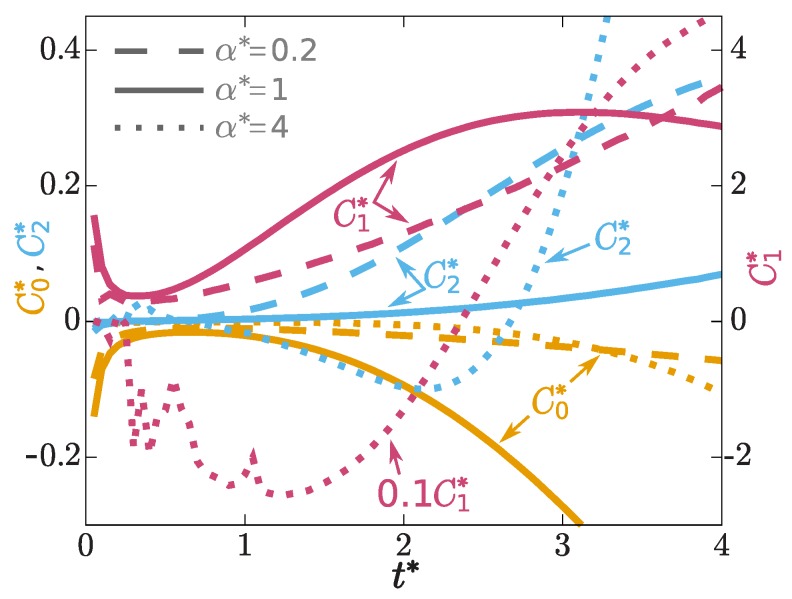
Scaled amplitudes $C_{0}^{*}=C_{0}\sqrt{m\omega /\hbar^{3}}$ (yellow), $C_{1}^{*}=C_{1}\sqrt{m\omega /\hbar^{3}}$ (violet) and $C_{2}^{*}=C_{2}\sqrt{m\omega^{3}/\hbar^{3}}$ (blue) of the functional approximations for $\tau_{\mathrm{exc}}$, $F_{v}$ and $F_{a}$, cf. Equations (8), (10) and (11), as a function of the scaled time $t^{*}=t\omega$ for three different values of the interaction length, as indicated. Note the different scales (left and right vertical axes). The data set $C_{1}^{*}$ ($\alpha^{*}=4$) has been scaled with a factor 0.1, as indicated, for a better visualization.

First we focus on the stress tensor. Splitting Equation (6) into ideal and excess parts is illustrated in Figure 2a–c. The relative importance of the excess contribution $\tau_{\mathrm{exc}}$ to the total stress depends on the value of the interaction length and it is most dominant for $\alpha^{*}=1$, which is comparable to the initial width of the density distribution. The symmetry and simple shape of $\tau_{\mathrm{exc}}$ suggests that ∂v/∂x is a meaningful variable to describe it. Hence, we approximate $\tau_{\mathrm{exc}}$ by

(8) $$\tau_{\mathrm{exc}}\left[n,v\right]=\int_{0}^{t}dt^{\prime }\int dx^{\prime }nK_{0}\left(x-x^{\prime },t-t^{\prime }\right)n^{\prime }\frac{\partial v^{\prime }}{\partial x^{\prime }},$$

where n≡n(x,t), $n^{\prime }\equiv n\left(x^{\prime },t^{\prime }\right)$ and $v^{\prime }\equiv v\left(x^{\prime },t^{\prime }\right)$, and $K_{0}$ is a convolution kernel that will in general be non-local in space and time. For simplicity, we use a Markovian and spatially local approximation for $K_{0}$. To compensate for the resulting lack of memory, we let the strength of the Markovian form depend explicitly on time *t*. Hence we replace $K_{0}\left(x-x^{\prime },t,t^{\prime }\right)$ in Equation (8) by $C_{0}\left(t\right)\delta \left(x-x^{\prime }\right)\delta \left(t-t^{\prime }\right)$, where the amplitude $C_{0}$ carries an explicit time dependence, which allows us to match the overall amplitude of the theoretical spatial profile to the exact numerical data. Figure 2a–c show that the theory captures the excess contribution to the stress tensor well for the different values of the interaction length considered here. The value of $C_{0}\left(t\right)$ at each time, see Figure 3, is determined by fitting (least squares) the convolution Equation (8) to the exact numerical solution.

The splitting of the internal force density into adiabatic and superadiabatic components is shown in Figure 2d–f. The superadiabatic force density is a prominent contribution to the total internal force density. The superadiabatic component opposes the adiabatic force density, hampering the spreading of the density peak. As we demonstrate below, the functional dependence on the velocity field is consistent with the interpretation of a viscous effect. For small interaction lengths, Figure 2d inset and Figure 2e, the spatial structure of $F_{\sup}$ is more complex with additional peaks that speed up the dynamics of the wings of the density profile. These findings motivate the mathematical structure of the approximate form of $F_{\sup}$, as a sum of two contributions,

(9) $$F_{\sup}\left[n,v,a\right]=F_{v}\left[n,v\right]+F_{a}\left[n,a\right],$$

where both terms are functionals of the density profile and of either the velocity or the acceleration profiles:

(10) $$\begin{array}{cc} F_{v}\left[n,v\right] & =\int_{0}^{t}dt^{\prime }\int dx^{\prime }nK_{1}\left(x-x^{\prime },t-t^{\prime }\right)n^{\prime }\frac{\partial^{2}v^{\prime }}{\partial {x^{\prime }}^{2}} \end{array}$$

(11) $$\begin{array}{cc} F_{a}\left[n,a\right] & =\int_{0}^{t}dt^{\prime }\int dx^{\prime }nK_{2}\left(x-x^{\prime },t-t^{\prime }\right)n^{\prime }\frac{\partial^{2}a^{\prime }}{\partial {x^{\prime }}^{2}} \end{array}$$

with $a^{\prime }\equiv a\left(x^{\prime },t^{\prime }\right)$. Note the structural similarity to Equation (8). As before, the convolution kernels $K_{1}$ and $K_{2}$ are in general non-local in space and time, but we use a Markovian and spatially local approximation here, and replace $K_{i}\left(x-x^{\prime },t-t^{\prime }\right)$, by $C_{i}\left(t\right)\delta \left(x-x^{\prime }\right)\delta \left(t-t^{\prime }\right)$, where the prefactor $C_{i}\left(t\right)$ depends explicitly on time and i=1,2. Equation (10) possesses a similar structure as the volume viscous forces appearing in the Navier-Stokes equation [31,51]. Figure 2d–f shows that the theory reproduces the shape of the superadiabatic force density very accurately. Both Equations (10) and (11) are important, as shown in the inset of Figure 2e. The amplitudes of both terms, $C_{1}\left(t\right)$ and $C_{2}\left(t\right)$, are again obtained by finding the best fit (least squares) of Equation (9) to the data. The results are shown in Figure 3.

## 4. Conclusions

In conclusion, we have presented a power functional approximation that satisfactorily describes superadiabatic forces in a prototypical time-dependent quantum system. We have demonstrated that the microscopic, position- and time-resolved acceleration gradient, $\nabla (\dot{J}/n)$, is a crucial field in the description of superadiabatic quantum effects. The velocity gradient ∇(J/n) is a further relevant variable, consistent with what is found in time-dependent current DFT, see, e.g., reference [22]. The relatively simple time dependence of the superadiabatic forces, Figure 3, suggest that power functional theory is a promising approach to investigate memory effects quantitatively. While Equations (8), (10) and (11) resembles the Vignale-Kohn approximation [9], in our formulation the acceleration gradient plays a prominent role; moreover, the superadiabatic contributions are obtained from a generating (power) functional [34]. Further work is required to explore the relationship of our approach to the time-dependent current DFT [9,10,12,16,17,18] in more depth. Given the vectorial character of the forces, a systematic investigation of memory effects and of the superadiabatic forces is required to go beyond the one-dimensional geometry considered here. The two-dimensional setup of reference [22] could be a promising candidate for carrying out such work.
